# Left Ventricular Diastolic Dysfunction in Dialysis Patients Assessed by Novel Speckle Tracking Strain Rate Analysis: Prevalence and Determinants

**DOI:** 10.1155/2012/963504

**Published:** 2012-05-09

**Authors:** Mihály K. de Bie, Nina Ajmone Marsan, André Gaasbeek, Jeroen J. Bax, Marc Groeneveld, Bas A. Gabreels, Victoria Delgado, Ton J. Rabelink, Martin J. Schalij, J. Wouter Jukema

**Affiliations:** ^1^Department of Cardiology, Leiden University Medical Center, 2300 RC Leiden, The Netherlands; ^2^Department of Nephrology, Leiden University Medical Center, 2300 RC Leiden, The Netherlands; ^3^Department of Nephrology, Medisch Centrum Haagland, 2125 VA, The Hague, The Netherlands; ^4^Department of Nephrology, Rijnland Ziekenhuis, Leiderdorp, The Netherlands

## Abstract

*Background*. Diastolic dysfunction is common among dialysis patients and is associated with increased morbidity and mortality. Novel echocardiographic speckle tracking strain analysis permits accurate assessment of left ventricular diastolic function, independent of loading conditions and taking all myocardial segments into account. The aim of the study was to evaluate the prevalence of diastolic dysfunction in chronic dialysis patients using this novel technique, and to identify its determinants among clinical and echocardiographic variables. *Methods*. Patients currently enrolled in the ICD2 study protocol were included for this analysis. Next to conventional echo measurements diastolic function was also assessed by global diastolic strain rate during isovolumic relaxation (SRIVR). *Results*. A total of 77 patients were included (age 67 ± 8 years, 74% male). When defined as E/SRIVR ≥236, the prevalence of diastolic dysfunction was higher compared to more conventional measurements (48% versus 39%). Left ventricular mass (OR 1.02, 95% CI 1.00–1.04, *P* = 0.014) and pulse wave velocity (OR 1.34, 95% CI 1.07–1.68, *P* = 0.01) were independent determinants of diastolic dysfunction. *Conclusion*. Diastolic dysfunction is highly prevalent among dialysis patients and might be underestimated using conventional measurements. Left ventricular mass and pulse wave velocity were the only determinants of diastolic dysfunction in these patients.

## 1. Introduction

In dialysis patients, both cardiovascular and noncardiovascular mortality are significantly increased as compared to the general population [1]. In particular, cardiovascular mortality contributes to ~40% of all-cause mortality in these patients, mainly due to sudden cardiac death [2]. Several parameters, such as left ventricular hypertrophy (LVH) and left ventricular (LV) systolic dysfunction, have been identified as independent predictors of (cardiovascular) outcome in dialysis patients. Next to that significant diastolic heart dysfunction, as assessed by tissue Doppler imaging (TDI), has also demonstrated significant incremental prognostic value for all-cause mortality and cardiovascular death [3]. Similar to the general population, diastolic heart failure in dialysis patients often exists without the presence of significant systolic heart failure [4, 5]. Therefore, accurate evaluation of LV diastolic dysfunction is crucial in the management and risk stratification of dialysis patients, especially in those with preserved ejection fraction. Particularly, LV diastolic function and its determinants might represent an important target for therapeutic options aimed at improving the abysmal prognosis of this group of dialysis patients with preserved ejection fraction.

Recently, LV global diastolic strain rate (SR) measurement by 2D echocardiography speckle-tracking analysis was demonstrated to be superior to the mitral annulus velocities measured by TDI, for the assessment of LV diastolic function, especially in patients with relatively preserved LV ejection fraction (EF) or with LV regional wall motion abnormalities. In particular, mitral early diastolic velocity (*E*)/SR ratio during LV isovolumetric relaxation (IVR) period showed to be highly correlated with the invasive measure of pulmonary capillary wedge pressure, and a cut-off value of 236 was able to identify patients with elevated LV filling pressure with high sensitivity and specificity [6].

The objective of this study was therefore to assess the prevalence of significant LV diastolic dysfunction using this novel echocardiographic technique in a group of dialysis patients with preserved LV systolic function. Furthermore, possible determinants of LV diastolic dysfunction, among several clinical and echocardiographic variables, were identified.

## 2. Methods

### 2.1. Patient Population and Protocol

All patients currently enrolled in the ICD2 study (ISRCTN20479861) were included in the current analysis. The rationale and protocol of this study have been previously reported [7]. Briefly, this study evaluates the effect of prophylactic implantable cardioverter defibrillator (ICD) in chronic dialysis patients (age 55–80 years). Patients meeting current ICD implantation criteria [8] and patients with severe comorbidities resulting in a life expectancy of less than one year are not considered for inclusion in the ICD2 study. The ICD2 study protocol has been approved by the local ethics committee, and all participating patients provided written informed consent.

All included patients underwent an extensive screening evaluation before randomization to the treatment or control arm of the study. In particular, patients on hemodialysis were evaluated on the day prior to a dialysis session. Information was obtained on medical history and medication use. A physical examination was performed, and EDTA and heparinized blood samples were collected. Transthoracic echocardiography was performed, including conventional and advanced measures of LV diastolic function. Finally, vascular stiffness was assessed by applanation tonometry.

### 2.2. Echocardiographic Evaluation

All patients were examined in the left lateral decubitus position with a commercially available ultrasound transducer and equipment (M3s probe, Vivid 7, GE Vingmed, Horton, Norway). The images were digitally stored for off-line analysis (EchoPAC version 110.0.0, GE Vingmed, Horten, Norway).

According to standard techniques, a complete 2D, color, pulse- and continuous-wave Doppler echocardiogram was performed. Using Simpson's biplane method [9], LV volumes and ejection fraction were assessed from apical 2- and 4-chamber views. LV mass was calculated by Devereux's formula using the M-mode of the parasternal long axis view, and indexed to body surface area [9]. Relative wall thickness (RWT) was calculated by the formula (2 × posterior wall thickness in diastole)/LV internal dimension in diastole [9].

As recommended by the American Society of Echocardiography, maximal left atrial (LA) volume was calculated using the ellipsoid model and indexed to body surface area [9].

Several conventional parameters of LV diastolic function were assessed. Transmitral E-wave velocity, E-wave deceleration time (DT), and late diastolic wave (*A*) velocity were measured applying pulse-wave Doppler (sample volume = 2 mm) at the tip of the mitral leaflets in the 4-chamber view. Furthermore, TDI was recorded with high frame rate (≥100 frames/second) from the apical 4-chamber view to assess myocardial velocities. Peak annular early diastolic velocity (*E*′) was measured in 2 annular LV segments (septal and lateral) and averaged to calculate the mean early diastolic velocity. The ratio *E*/*E*′ was calculated, as a validated estimate of LV filling pressure, and significant LV diastolic dysfunction was defined as *E*/*E*′ ≥ 15.0 [10, 11].

**Figure 1:**
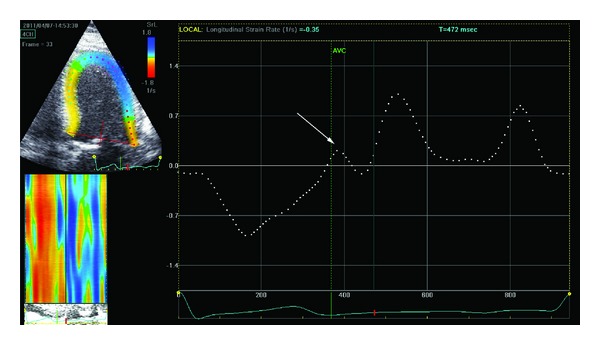
Evaluation of peak strain rate during left ventricular isovolumetric relaxation period SR_IVR_ from the apical 4-chamber view. First, a region of interest which includes the entire left ventricular wall is obtained (left-upper panel). The software consequently displays the changes in longitudinal strain rate over the cardiac cycle (white dotted line). Left ventricular isovolumetric relaxation occurs in diastole, starting immediately after closure of the aortic valve and terminating with the beginning of the diastolic early (*E*) wave. The peak strain rate value during the isovolumetric relaxation period (SR_IVR_) is consequently measured (indicated by the white arrow). The green dotted line indicates the timing of the aortic valve closure (AVC).

Advanced measures of LV diastolic function were obtained by 2D speckle tracking SR analysis. This imaging technique allows for the assessment of LV myocardial deformation by tracking natural acoustic markers (speckles) in a frame-to-frame basis within the cardiac cycle. The speckles are visible in the standard gray-scale 2D images and are equally distributed within the myocardium. Strain rate during isovolumic relaxation period (SR_IVR_) was measured as previously described [6, 12]. Briefly, the endocardial border was manually traced on LV apical 4-, 2-, and 3-chamber views and the region-of-interest width was adjusted to include the entire myocardium. The images were optimized in order to obtain the highest possible frame rate. Longitudinal SR curves were generated for each apical view, and peak global SR_IVR_ was measured and averaged from the 3 apical views (Figure 1). The ratio of *E*/SR_IVR_ was calculated as a novel index of LV filling pressure. As previously proposed, a *E*/SR_IVR_ ≥ 236 was used to identify patients with significant LV diastolic dysfunction [6].

### 2.3. Assessment of Vascular Stiffness

Vascular stiffness was assessed noninvasively with applanation tonometry using a SphygmoCor system (SphygomoCor, Atcor Medical, Sydney, Australia). All measurements were performed in a quiet, temperature-controlled clinical research laboratory. Measurements were performed following a 10-minute rest in supine position, after a state of constant heart rate and blood pressure was reached.

The aortic pulse wave velocity (PWV) was defined as the distance traveled by the pulse wave between 2 recording sites, divided by the passage time. Using sequential tonometry with simultaneous electrographic gating, pulse waves were recoded at the common carotid artery and the femoral artery. Following the measurements, the system software calculated the PWV. This semiautomatic method to assess PWV has been previously validated [13].

### 2.4. Statistical Analysis

When normally distributed (as assessed by the Kolmogorv-Smirnov test), continuous variables are expressed as mean ± SD and as median (25th and 75th percentiles *Q*_1_, *Q*_3_) when nonnormally distributed. Continuous data were compared using unpaired *t*-test when normally distributed and using the Mann-Whitney *U*-test when non-normally distributed. Categorical data are expressed as frequencies and percentages and were compared using *χ* ^2^ test. Correlation between *E*/SR_IVR_ and baseline parameters was assessed using Spearsmans' rank correlation coefficient. Multiple logistic regression analysis was performed in order to establish the relationship between different parameters and the presence of LV diastolic dysfunction. For all tests, a *P* value < 0.05 was considered statistically significant. All analyses were performed in SPSS version 18.0.

## 3. Results

A total of 89 patients currently enrolled in the ICD2 trial were included in this analysis. Eight patients were excluded due to image quality not sufficient for strain analysis, and 4 patients were excluded since PWV assessment was not feasible. Baseline clinical characteristics of the remaining 77 patients are summarized in Table 1. Mean age of the included patients was 67.2 ± 7.8 years. Patients were predominantly male (74%), and the majority of patients were on hemodialysis (the remaining patients were on peritoneal dialysis) with an average dialysis vintage of approximately 27 months (median 16 months).

In these patients, echocardiography (Table 2) revealed a relatively preserved LV size and EF, but a significant increase of LV mass. Average *E*/*E*′ was 14.9 ± 6.3, and an *E*/*E*′ ≥ 15.0, as index of significant LV dysfunction, was documented in 30 (39%) patients. However using more advanced measures of LV diastolic function, the prevalence of significant LV diastolic dysfunction was higher: a total of 37 (48%) patients showed an *E*/SR_IVR_ ≥ 236.

### 3.1. Comparison of Patients with and without Significant LV Diastolic Dysfunction

Clinical characteristics for patients with (*E*/SR_IVR_ ≥ 236) and without significant LV diastolic dysfunction (*E*/SR_IVR_ < 236) are summarized in Table 1. These groups were comparable with regard to age and gender, but the prevalence of hypertension (97% versus 77%, *P* < 0.05) and diabetes (43% versus 20%, *P* < 0.05) was higher in patients with LV diastolic dysfunction. These patients were also on hemodialysis more often (73% versus 47.5%, *P* < 0.05). Differences in dialysis vintage and history of myocardial infarction did not reach statistical significance.

These groups showed also significant differences in echocardiographic characteristics (Table 2). LV and LA volumes were significantly larger in patients with LV diastolic dysfunction, and LV mass index (LVMI) was significantly higher (148 ± 48 g/m^2^ versus 111 ± 34 g/m^2^). Vascular stiffness was also significantly increased in patients with diastolic dysfunction, resulting in an increased PWV as compared to patients without LV diastolic dysfunction (12.3 ± 3.2 m/s versus 9.6 ± 2.8 m/s, *P* < 0.05).

### 3.2. Determinants of LV Diastolic Dysfunction

Correlation analysis revealed a significant positive relationship between LVMI and *E*/SR_IVR_ and between PWV and *E*/SR_IVR_ (*r* = 0.43, *P* = 0.001 and *r* = 0.31, *P* = 0.007, resp.). In addition, in order to identify potential determinants of LV diastolic dysfunction, logistic uni- and multivariate regression analyses were performed (Table 3). At the univariate analysis, hemodialysis (OR 2.98, 95% CI 1.15–7.75, *P* = 0.025), hypertension (OR 10.45, 95% CI 1.26–87.16, *P* = 0.003), diabetes mellitus (OR 3.05, 95% CI 1.11–8.38, *P* = 0.031), PWV (OR 1.28, 95% CI 1.08–1.50, *P* = 0.004), RWT (OR 1.04, 95% CI 1.01–1.08, *P* = 0.022), and LVMI (OR 1.022, 95% CI 1.01–1.04, *P* = 0.001) were significant predictors for the presence of LV diastolic dysfunction. The multivariate model included all parameters with a *P* value < 0.20 in the univariate analysis and demonstrated that PWV (OR 1.34, 95% CI 1.07–1.68, *P* = 0.015) and LVMI (OR 1.02, 95% CI 1.00–1.04, *P* = 0.014) were the only independent determinants of significant LV diastolic dysfunction.

## 4. Discussion

The key findings of this study can be summarized as follows: using LV global diastolic SR measurement, the prevalence of LV diastolic dysfunction in dialysis patients with preserved LVEF was approximately 50%. Using conventional echocardiographic methods, this prevalence was lower, indicating a possible underestimation when applying these techniques. In addition, LVMI and PWV were the only independent determinants of LV diastolic dysfunction in this patient group.

### 4.1. Cardiovascular Mortality in Dialysis Patients

Cardiovascular disease largely contributes to the poor outcome in dialysis patients, and sudden cardiac death especially has been put forward as an important cause of mortality in this patient group. It has been reported that sudden cardiac death accounts for almost 60% of cardiovascular mortality and is the cause of death in approximately 25% of dialysis patients [2, 14, 15]. The mechanisms underlying sudden cardiac death in this patient group are complex and not yet completely understood. Next to the traditional cardiovascular risk factors, such as ischaemic heart disease [16], many other factors have also been reported to be associated with cardiovascular death in dialysis patients, including LVH, abnormalities in myocardial ultrastructure and function, interstitial fibrosis, and sympathetic overactivity [14, 17].

Recently, it has been demonstrated that LV diastolic dysfunction provides independent and additional prognostic value for long-term mortality and cardiovascular death in patients with end-stage renal disease, above and beyond that of LVM and LVEF [3]. Therefore, identifying patients with LV diastolic dysfunction might help in developing treatment strategies and in selecting patients most likely to benefit from these strategies. Given the possible underestimation of diastolic dysfunction using more conventional echocardiographic measurements, this novel technique would probably allow for more optimal patient selection for these treatment strategies. Finally, preventing the development of LV diastolic dysfunction might also improve outcome and reduce the incidence of cardiovascular death in dialysis patients.

### 4.2. Assessment of LV Diastolic Function

Recent studies evaluating LV diastolic function in dialysis patients used the ratio of early mitral diastolic velocity to early diastolic velocity (*E*/*E*′ ratio) [3, 18]. Although it is a clinically useful method to assess LV diastolic function, the measurement of *E*′ has a number of limitations which can affect its accuracy. Firstly, this approach is an approximation of LV global function, assuming that a single or multiple site(s) represent global LV relaxation. However, it is known that even in patients without segmental dysfunction, LV regional differences exist. Particularly in dialysis patients, significant LV diastolic dyssynchrony has been described to be often present [19].

Another important limitation of this approach is the potential effect of LA pressure. Since *E*′ occurs during the early phase of LV filling, not only LV relaxation, but also LA pressure has an important impact on its value [20, 21]. This limitation is particularly important in dialysis patients, given the high prevalence of hypertension and chronic fluid overload in these patients.

Conversely, global LV diastolic SR might overcome all the abovementioned limitations. This measurement reflects in fact the performance of all LV segments, it is load independent and accounts for the initial LV size. Furthermore, when measured during the IVR period (when the mitral valve is still closed) and expressed in a ratio with the transmitral E wave, it takes into account the mean LA pressure [6]. Recently, Wang et al. demonstrated that *E*/SR_IVR_ can be used to estimate LV filling pressures, with higher sensitivity and specificity as compared to *E*/*E*′, particularly in patients with preserved LVEF and in those with regional dysfunction [6]. However, the measurement of global SR_IVR_ requires the presence of adequate apical views, and therefore it is not feasible in patients with suboptimal apical windows. In the present study, this measurement was feasible in 92% of the patients.

### 4.3. Determinants of LV Diastolic Dysfunction

Among the clinical and echocardiographic variables assessed in this study, LVMI and aortic stiffness, assessed with PWV, were the only independent predictors for the presence of LV diastolic dysfunction. Therapies preventing further progression of LVMI and/or aortic stiffness might therefore prevent and/or limit the development of LV diastolic dysfunction.

Several therapeutic strategies have shown beneficial effect on LVH (limiting progression or even inducing regression), including angiotensin-converting enzyme inhibitor (ACEi) [22], angiotensin reuptake blocker (ARB) therapy, and increasing dialysis frequency (nocturnal) [23, 24]. However, their use has not been (yet) associated with a statistically significant reduction in fatal and nonfatal cardiovascular events.

Arterial stiffening occurs normally with aging and also correlates with the prevalence of atherosclerosis [25]. For patients with end-stage renal disease, additional mechanisms are probably responsible for increased arterial stiffening, namely, the extensive calcification of the vessel wall, resulting in both heavily calcified atherosclerotic plaques and calcification of the muscular media layer of the vessel wall [26]. Therapeutic strategies aiming at reducing progression of vascular calcification could therefore prevent further progression of arterial stiffening and thereby the development of LV diastolic dysfunction. Current strategies that are being investigated in this regard focus on inhibiting the effects of secondary hyperparathyroidism with phosphate binders and calcimimetics [27, 28]. Vascular stiffening may also be improved using antihypertensive medication (ACEi, ARB, B blockers) [29]; however, due the small sample sizes and the selected patient groups in which the studies have been conducted, these results cannot be generalized to dialysis patients.

Advanced glycation end product cross-link breakers also have emerged as a potential therapeutic strategy, and it has been demonstrated that cross-link breakers improve arterial compliance, PWV, and pulse pressure in a selected group of patients [30].

### 4.4. Study Limitations

Given the lack of invasive measurements, this study does not allow for definitive conclusions regarding the accuracy of *E*/SR_IVR_ for the identification of significant LV diastolic dysfunction. It should be noted however that Wang et al. recently demonstrated in a validation study that *E*/SR_IVR_ has significantly higher specificity and sensitivity as compared to more conventional measurements for the estimate of LV filling pressures. Furthermore, this study was primarily designed to assess and describe the prevalence of LV diastolic dysfunction according to an advanced echocardiographic measurement and moreover to establish its clinical determinants, rather than to compare it to more conventional echocardiographic techniques. The patients used in this analysis are patients with a life expectation of at least 1 year, given the nature of the ICD2 study protocol. Therefore the “sickest” patients are being excluded, and therefore the prevalence of LV diastolic dysfunction might be underestimated. Furthermore, due to the relatively low number of patients and relatively short period of follow-up in the ICD2 study, long-term outcome data could not be included. Before the true additional value of this novel technique can be established, also long-term outcome data should be analyzed in future studies.

## 5. Conclusion

In dialysis patients with preserved LVEF, the prevalence of LV diastolic dysfunction assessed by global LV SR is relatively high and might be underestimated when using conventional echocardiographic techniques. In these patients, LV mass and PWV are independent predictors of LV diastolic dysfunction and could serve as possible therapeutic targets.

## Figures and Tables

**Table 1:** Baseline clinical characteristics of the total patient population and divided according to the presence (*E*/SR_IVR_ ≥ 236) or not (*E*/SR_IVR_ < 236) of significant left ventricular diastolic dysfunction.

	All patients (*n* = 77)	*E*/SR_IVR_ < 236 (*n* = 40)	*E*/SR_IVR_ ≥ 236 (*n* = 37)
Age (yrs.)	67.2 ± 7.8	66.9 ± 8.3	67.7 ± 7.4
Male gender	57 (74.0%)	28 (70.0%)	29 (78.4%)
Hemodialysis	46 (59.7%)	19 (47.5%)	27 (73.0%)*
Dialysis vintage (months)	16 [9,34]	12 [8,26]	21 [10,52]
NYHA class			
I	52 (68.5%)	30 (75.0%)	22 (59.5%)
II/III	25 (31.5%)	10 (25.0%)	15 (40.5%)
Hypertension	67 (87.0%)	31 (77.5%)	36 (97.3%)*
BSA	1.93 ± 0.21	1.93 ± 0.20	1.92 ± 0.21
Diabetes mellitus	24 (31.2%)	8 (20.0%)	16 (43.3%)*
Myocardial infarction	21 (27.3%)	9 (22.5%)	12 (32.4%)
Hemoglobin (mmoL/L)	7.6 ± 0.8	7.6 ± 0.7	7.7 ± 0.9
Creatinine (*μ*mol/L)	649 ± 203	650 ± 230	648 ± 174
Calcium (mmoL/L)	2.37 ± 0.17	2.4 ± 0.19	2.4 ± 0.17
Phosphate (mmoL/L)	1.49 ± 0.31	1.5 ± 0.3	1.5 ± 0.3
*β*-blocker	43 (55.8%)	22 (55.0%)	21 (56.8%)
ACEI/ARB	41 (53.2%)	22 (55.0%)	19 (51.4%)
Statins	55 (71.4%)	28 (70.0%)	27 (72.3%)
PWV (m/s)	10.9 ± 3.3	9.6 ± 2.8	12.3 ± 3.3*

*P < 0.05 between patients with *E*/SR_IVR_ < 236 and patients with *E*/SR_IVR_ ≥ 236.

ACEI/ARB: angiotensin-converting enzyme inhibitors or receptor blockers; BSA: body surface area; NYHA: New York Heart Association; PWV: pulse wave velocity.

**Table 2:** Echocardiographic characteristics of the total patient population and divided according to the presence (*E*/SR_IVR_ ≥ 236) or not (*E*/SR_IVR_ < 236) of significant left ventricular diastolic dysfunction.

	All patients (*n* = 77)	*E*/SR_IVR_ < 236 (*n* = 40)	*E*/SR_IVR_ ≥ 236 (*n* = 37)
LVEDV (mL)	107 ± 40	97 ± 38	118 ± 42*
LVESV (mL)	52 ± 26	46 ± 22	60 ± 28*
LVEF (%)	52% ± 7	54% ± 6	51% ± 8
LA volume (mL/m^2^)	26.8 ± 12.2	22.7 ± 10.9	31.3 ± 12.1*
LVMI (g/m^2^)	129 ± 45	111 ± 34	148 ± 48*
RWT	0.53 ± 0.15	0.49 ± 0.13	0.57 ± 0.15*
E (cm/s)	0.75 ± 0.25	0.71 ± 0.23	0.77 ± 0.26
*E*/*A* ratio	0.88 ± 0.33	0.86 ± 0.33	0.90 ± 0.34
DT (ms)	261 ± 81	267 ± 78	255 ± 84
*E*/*E*′ ratio	14.9 ± 6.3	12.1 ± 4.8	17.9 ± 6.3*
SR_IVR_	203 [145,353]	149 [107,189]	354 [304,457]*

*P < 0.05 between patients with *E*/SR_IVR_ < 236 and patients with *E*/SR_IVR_ ≥ 236.

A: late diastolic transmitral flow velocity; DT: deceleration time; *E*: early diastolic transmitral inflor velocity; *E*′: peak annular early diastolic velocity; LA: left atrial; LVEDV: left ventricular end diastolic volume; LVEF: left ventricular ejection fraction; LVESV: left ventricular end systolic volume; LVMI: left ventricular mass index; RWT: relative wall thickness; SR_IVR_: strain rate during isovolumic relaxation.

**Table 3:** Uni- and multivariate logistic regression analysis to predict the presence of LV diastolic dysfunction (defined as *E*/SR_IVR_ ≥ 236).

		Univariate			Multivariate	
	HR	95% CI	*P*-value	HR	95% CI	*P* value
Age	1.013	0.96–1.07	0.65			
Male gender	1.56	0.55–4.37	0.40			
Hemodialysis	2.98	1.15–7.75	0.025	2.64	0.75–9.21	0.13
Dialysis vintage (months)	1.016	1.00–1.04	0.085	1.013	0.99–1.03	0.22
Hypertension	10.45	1.25–87.16	0.03	4.91	0.46–51.83	0.19
DM	3.05	1.11–8.38	0.031	2.40	0.57–10.08	0.23
MI	1.65	0.60–4.55	0.33			
LVMI	1.022	1.01–1.04	0.001	1.02	1.00–1.04	0.014
RWT	1.04	1.01–1.08	0.022	0.99	0.94–1.04	0.652
PWV	1.35	1.13–1.62	0.001	1.34	1.07–1.68	0.010

DM: diabetes mellitus; LVMI: left ventricular mass index; MI: myocardial infarction; PWV: pulse wave velocity; RWT: relative wall thickness.
